# Congenital syndactyly in cattle: four novel mutations in the low density lipoprotein receptor-related protein 4 gene (*LRP4*)

**DOI:** 10.1186/1471-2156-8-5

**Published:** 2007-02-23

**Authors:** Cord Drögemüller, Tosso Leeb, Barbara Harlizius, Imke Tammen, Ottmar Distl, Martin Höltershinken, Arcangelo Gentile, Amandine Duchesne, André Eggen

**Affiliations:** 1 Institute of Genetics, Vetsuisse Faculty, University of Berne, Bremgartenstrasse 109a, 3001 Berne, Switzerland; 2 Institute for Animal Breeding and Genetics, University of Veterinary Medicine Hannover, Bünteweg 17p, 30559 Hannover, Germany; 3 Centre for Advanced Technologies in Animal Genetics and Reproduction (ReproGen), Faculty of Veterinary Science, The University of Sydney, Camden NSW 2570, Australia; 4 Clinic for Cattle, University of Veterinary Medicine Hannover, Bünteweg 17p, 30559 Hannover, Germany; 5 Veterinary Clinical Departement, University of Bologna, Via Tolara di Sopra 50, 40064 Ozzano dell'Emilia (Bologna), Italy; 6 INRA, UR339 Laboratoire de Génétique Biochimique et de Cytogénétique, 78350 Jouy-en-Josas, France

## Abstract

**Background:**

Isolated syndactyly in cattle, also known as mulefoot, is inherited as an autosomal recessive trait with variable penetrance in different cattle breeds. Recently, two independent mutations in the bovine *LRP4 *gene have been reported as the primary cause of syndactyly in the Holstein and Angus cattle breeds.

**Results:**

We confirmed the previously described *LRP4 *exon 33 two nucleotide substitution in most of the affected Holstein calves and revealed additional evidence for allelic heterogeneity by the identification of four new *LRP4 *non-synonymous point mutations co-segregating in Holstein, German Simmental and Simmental-Charolais families.

**Conclusion:**

We confirmed a significant role of *LRP4 *mutations in the pathogenesis of congenital syndactyly in cattle. The newly detected missense mutations in the *LRP4 *gene represent independent mutations affecting different conserved protein domains. However, the four newly described *LRP4 *mutations do still not explain all analyzed cases of syndactyly.

## Background

Many inherited malformations of domestic animals are analogous to human hereditary anomalies and have proven to be valuable animal models for the investigation of the pathogenesis of rare human phenotypes with identical molecular basis [1].

Isolated congenital syndactyly in cattle only affecting the digits, also known as mulefoot, refers to the fusion or non-division of the two developed digits of the bovine foot [2]. The variable expressed syndactyly phenotype in cattle is most often seen in the front feet, but all four feet underlying a right-left and front-rear gradient may be involved [3]. The bovine syndactyly consists mainly of pairs of horizontally synostotic phalanges and adaptive structural changes develop proximal to the fused digits [2]. Bovine syndactyly has been shown to segregate as a monogenic recessive trait with incomplete penetrance in many breeds of cattle in many countries (OMIA: 000963). Genetic mapping located the syndactyly locus on cattle chromosome 15 [4]. This bovine chromosome region is homologous to a segment of mouse chromosome 2 containing the low density lipoprotein receptor-related protein 4 gene (*Lrp4*), alternatively designated as multiple epidermal growth factor-like domains 7 gene (*Megf7*). Homozygous *Lrp4*-deficient mice are growth-retarded, with fully penetrant polysyndactyly in their fore and hind limbs [5]. Positional cloning of two recessive mutations of the mouse that cause polysyndactyly (*dan *and *mdig*) showed that the *Lrp4 *gene plays an essential role in the process of digit differentiation in mammalian species [6]. Other members of the low density lipoprotein receptor gene family have been shown to regulate intracellular signaling cascades [7]. In humans, syndactyly represents the most common congenital malformation of the hand and is characterized by the apparent fusion of soft tissue of the fingers and toes with or without bony fusion [8]. Syndactyly may occur as an isolated malformation or as part of a syndrome. Until now the two human genes *HOXD13 *and *GJA1 *were identified harboring causative dominant mutations for isolated syndactyly types II (OMIM: 186000) and III (OMIM: 186100), respectively. However, while clinical studies in these human defects revealed variable phenotypical expression, the establishment of precise genotype-phenotype correlations for limb malformations is difficult and the molecular genetic basis of numerous human cases of syndacytly is still unknown [8].

Recently, two independent causative mutations in the bovine *LRP4 *ortholog were identified in the predominantly affected cattle breeds of Holstein and Angus [9,10]. A homozygous *LRP4 *exon 33 substitution of two consecutive nucleotides (c.4863_4864delCGinsAT) was observed in 36 affected Holstein individuals, which lead to amino acid changes at two *LRP4 *codons (p. [Asn1621Lys; Gly1622Cys]) affecting a conserved EGF-like protein domain [9]. In the two reported affected Angus cattle a homozygous *LRP4 *single nucleotide substitution at the first base of intron 37 (c.5385+1G>A) disrupted the 5'splice site, which introduced aberrant splicing of intron 36 and lead to a truncated translation product lacking the normal N-terminal cytoplasmic domain [10]. The finding that mulefoot is caused by mutations within the same gene in both breeds is in agreement with earlier findings, where affected calves had experimentally been produced by mating of heterozygous Holstein and Angus cattle [11].

The aim of this study was to screen the bovine *LRP4 *gene for co-segregating mutations in sixteen animals from different breeds affected by congenital syndactyly.

## Results

Based on the recent identification of bovine *LRP4 *mutations causing congenital syndactyly this gene was studied in sixteen new mulefoot cases of four cattle families from three different breeds for co-segregating functional *LRP4 *sequence polymorphisms. The results are summarized in Table 1.

**Table 1:** Summary of *LRP4 *mutations described within this study.

**Family**	***LRP 4 *exon**	**Genomic DNA sequence change**	**LRP4 protein sequence change**	**Affected LRP4 protein domain**	**Predicted consequences**	
					**Polyphen**	**SIFT**
Holstein I/Crossbred	33	c.4863_4864delCGinsAT	p. [Asn1621Lys; Gly1622Cys]	LDL-type EGF-like	probably damaging	not tolerated
Holstein II	33	c.4940C>T	p.Pro1647Lys	LDL-type EGF-like	possibly damaging	not tolerated
Simmental	3	c.241G>A	p.Gly81Ser	LDL receptor class A 2	benign	tolerated
Simmental	26	c.3595G>A	p.Gly1199Ser	LDL receptor class B 13	possibly damaging	not tolerated
Crossbred	20	c.2719G>A	p.Gly907Arg	LDL receptor class B 8	probably damaging	not tolerated

The putative consequences of the polymorphisms on the modified proteins were analyzed using PolyPhen [13] and SIFT [14]. These programs are sequence homology-based tools that sort intolerant from tolerant amino acid substitutions and predict whether an amino acid substitution in a protein has a possible phenotypic effect. Since PolyPhen considers only human protein sequences, the bovine mutations were investigated in the context of the human LRP4 protein sequence (SwissProt Accession No. O75096).

### Holstein family I

**Figure 1:**
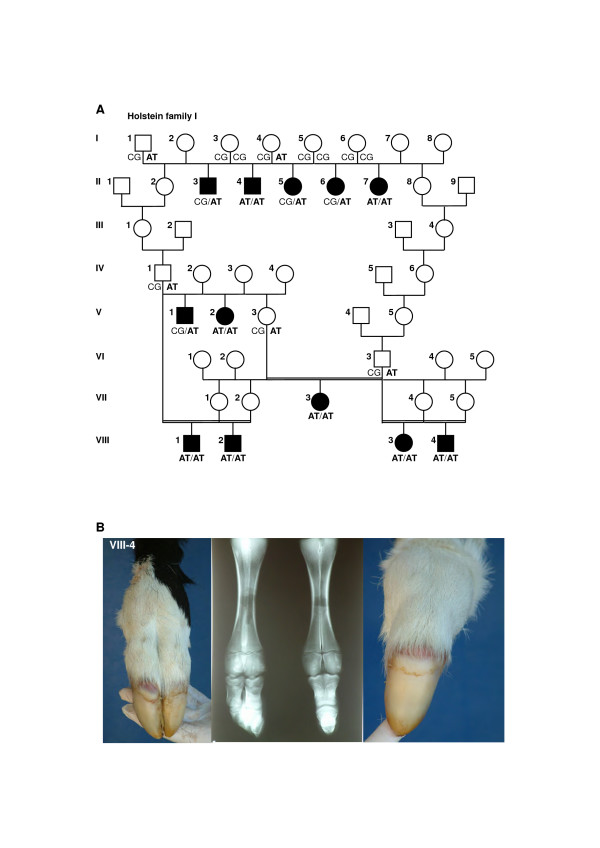
**Pedigree and *LRP4 *c.4863_4864delCGinsAT genotypes (A) and phenotype example (B) in Holstein family I**. (A) Pedigree chart of the Holstein family I used in this study. Animals affected with syndactyly are shown as solid black symbols. Samples from animals with genotypes for *LRP4 *c.4863_4864delCGinsAT were available for the molecular genetic analyses. Notice that the genotypes do not always fit to the phenotypes assuming a recessive mode of inheritance. (B) Italian Holstein calf (VIII-4) with two affected syndactylous forefeet. Phenotypic data from the eight affected animals of generation II to VII were reported before [3].

For eight German Holstein calves the phenotypes of one to four affected feet were previously described [3]. In the three Italian cases we observed only affected front feet, a single case with an affected right forefoot (VIII-2; Figure 1A) and two cases with two variably affected forefeet (VIII-3 and 4; Figure 1A and 1B). The new case from the German Holstein population showed three syndactylous feet, both forefeet and the right hindfoot (VIII-1; Figure 1A). Within this family eight out of twelve syndactyly affected animals were homozygous for the non-conservative substitution of two consecutive nucleotides in exon 33 (c.4863_4864delCGinsAT). Three available sires, two available dams and five paternal half-sibs of these eight calves showed heterozygosity for this exon 33 polymorphism, respectively (Figure 1A). For these animals no further exonic or splice site affecting sequence polymorphisms could be identified in the examined *LRP4 *exons. In contrast, the remaining four syndactylous calves, originating from the originally described German Holstein family [3], showed only a single copy of the c.4863_4864delCGinsAT allele. These four cases obtained the mutated exon 33 allele from their fathers as the three available dams of the four cases were homozygous for the wildtype allele at the c.4863_4864delCGinsAT polymorphism (Figure 1A). A comparison of all generated sequences for these four affected calves revealed three silent mutations: c.756T>C in exon 7, c.4269C>T in exon 29 and c.4749C>T in exon 32, respectively. Two affected calves were individually heterozygous at the exon 7 and exon 29 SNP, respectively, and inherited the respective mutated allele from their heterozygous dams. All four calves and the two dams were heterozygous at the exon 32 SNP. The three newly detected SNP were genotyped in 48 unrelated Holstein sires and showed minor allele frequencies for the mutated alleles of 0.33 (c.756T>C), 0.16 (c.4269C>T), and 0.48 (c.4749C>T), respectively.

### Holstein family II

**Figure 2:**
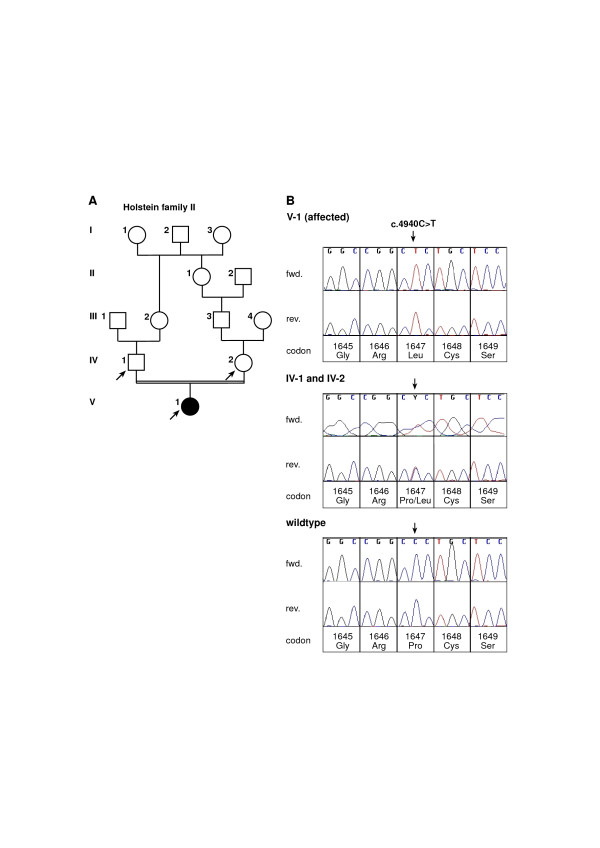
**Pedigree (A) and electropherograms (B) showing a mutation in the bovine *LRP4 *gene detected in Holstein family II**. (A) Pedigree chart of the Holstein family II used in this study. Animals affected with syndactyly are indicated as solid black symbols. Samples from animals indicated by arrows were available for the molecular genetic analyses. Phenotypic data from the other animals were obtained from the farmers records. (B) Sequence analysis of genomic DNA of the investigated animals. An arrow denotes the position of the point mutation. Note that the affected animal (V-1) is homozygous for the c.4940C>T mutation while its parents (IV-1 and IV-2) are heterozygous carriers of this mutation. Numbering of nucleotides and codons is according to the open reading frame of the cDNA sequence as deposited in GenBank (GenBank accession no. DQ462703).

The affected female calf showed a single syndactylous right forefoot. The pedigree records of the parents indicate a consanguineous mating of relatives with a common male ancestor resulting in an inbreeding coefficient of 1.56 % for the affected calf (Figure 2A). The affected calf in this family was found to be homozygous for a novel *LRP4 *SNP at position 103 of exon 33 (c.4940C>T) which was also identified in heterozygous state in both parents (Figure 2B). This point mutation predicts the exchange of proline to lysine at residue 1647 of the LRP4 protein (p.Pro1647Lys). No further exonic or intronic SNP could be observed in the three examined family members. The c.4940C>T SNP was not observed in 48 unrelated Holstein sires.

### Simmental family

**Figure 3:**
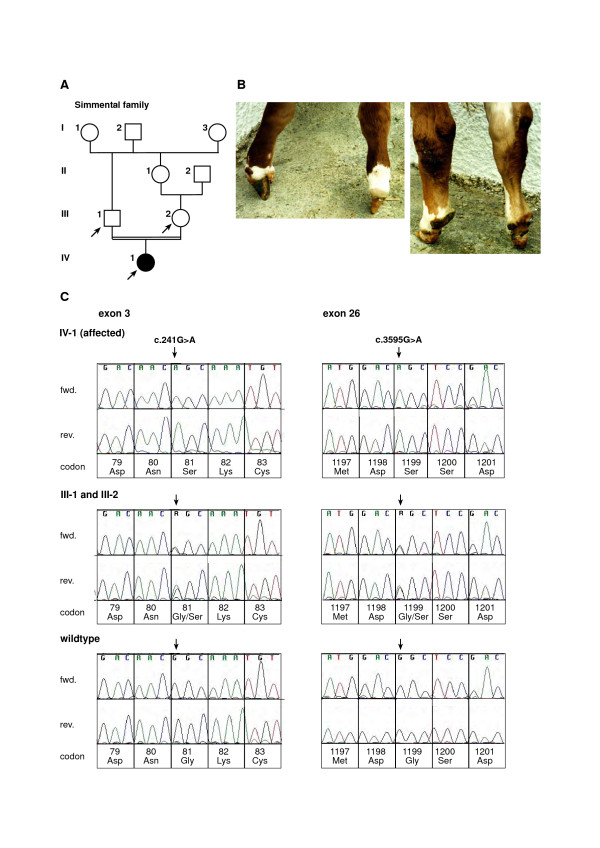
**Pedigree (A) and phenotype (B) and electropherograms (C) showing a mutation in the bovine *LRP4 *gene detected in Simmental family**. (A) Pedigree chart of the Simmental family used in this study. Animals affected with syndactyly are indicated as solid black symbols. Samples from animals indicated by arrows were available for the molecular genetic analyses. Phenotypic data from the other animals were obtained from the owners records. (B) German Simmental calf (IV-1) affected with syndactyly. Forefeet (left) and hindfeet (right). (C) Sequence analysis of genomic DNA of the investigated animals. An arrow denotes the position of the point mutation. Note that the affected animal (IV-1) is homozygous for the c.241G>A and the c.3595G>A mutations, respectively, while its parents (III-1 and III-2) show the heterozygous genotypes. Numbering of nucleotides and codons is according to the open reading frame of the cDNA sequence as deposited in GenBank (GenBank accession no. DQ462703).

All four feet of the female Simmental calf showed syndactyly (Figure 3B). The reconstruction of the relationship between the parents indicates a consanguineous mating of relatives with a common male ancestor resulting in an inbreeding coefficient of 6.25 % for the affected calf (Figure 3A). Within this family we observed two polymorphic exonic *LRP4 *sequence sites which are both located within CpG dinucleotides and no intronic SNP. The affected calf in this family was found to be homozygous for a novel *LRP4 *SNP at position 42 of exon 3 (c.241G>A) which was also identified in heterozygous state in both parents (Figure 3C). This point mutation predicts the exchange of glycine to serine at residue 81 of the LRP4 protein (p.Gly81Ser). The second SNP (c.3595G>A) was located at position 59 of exon 26 and predicts an exchange of glycine to serine at residue 1199 of the LRP4 protein (p.Gly1199Ser). This second SNP was homozygous in the affected and heterozygous in the parents as well (Figure 3C). Sequencing of 48 unrelated control Simmental animals showed that these SNP are not present in cattle free of syndactly.

### Crossbred family

**Figure 4:**
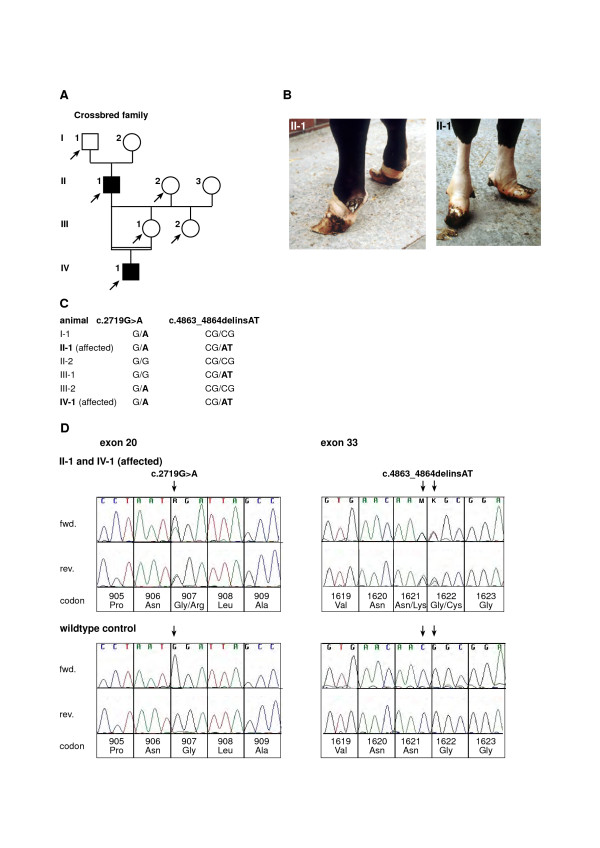
**Pedigree (A) and phenotype (B) and electropherograms (C) showing a mutation in the bovine *LRP4 *gene detected in crossbred family**. (A) Pedigree chart of the Crossbred family used in this study. Animals affected with syndactyly are shown as solid symbols. Samples from animals indicated by arrows were available for the molecular genetic analyses. Phenotypic data from the other animals were obtained from the owners records. (B) Adult bull (II-1) with syndactyly. Forefeet (left) and hindfeet (right). (C) Genotypes at c.2719G>A and c.4863_4864delCGinsAT for the six available animals. Note that both affected animals are heterozygous for both mutations. (D) Sequence analysis of genomic DNA of affected and a unrelated wildtype control animals. An arrow denotes the position of the mutations. Numbering of nucleotides and codons is according to the open reading frame of the cDNA sequence as deposited in GenBank (GenBank accession no. DQ462703).

The syndactyly phenotype of both affected family member was quite similar as all four feet showed fused phalanges with single hoof-like structure (Figure 4B). The pedigree analysis revealed that the first affected bull (II-1) was produced by mating of a Simmental × Charolais artificial insemination sire (I-1) to a German Holstein cow (I-2) (Figure 4A). Then, an experimental backcross of the affected sire to one of his daughters (III-1) was performed. This consanguineous mating produced the second affect male calf (IV-1) with an inbreeding coefficient of 25 % (Figure 4A). The molecular genetic analysis revealed two exonic and no intronic *LRP4 *polymorphisms. A newly detected SNP at position 107 of exon 20 (c.2719G>A) predicts an amino acid exchange at residue 907 of the LRP4 protein (p.Gly907Arg). The second sequence variation observed within this family was previously reported in Holstein cattle, a c.4863_4864delCGinsAT polymorphism altering the LRP4 protein sequence. The c.2719G>A SNP segregates in the four generation pedigree, where two affected and two healthy members were heterozygous, respectively (Figure 4C). Both affected individuals and one mother (III-1) were also heterozygous for the c.4863_4864delCGinsAT polymorphism, respectively (Figure 4C). The c.2719G>A SNP was not observed in 16 unrelated controls belonging to the Simmental, Holstein and Charolais breed, respectively.

## Discussion

This study indicates that congenital syndactyly may occur in different breeds of cattle due to mutations in *LRP4*. All reported family histories are compatible with autosomal recessive inheritance of bovine syndactyly. Also the known variability in the phenotypic expression characterized by the variable number of affected feet and the gradually different fusion of bones per foot could be confirmed by the reported sixteen cases. Most interesting, four novel mutations in the bovine *LRP4 *gene were detected which possibly lead to syndactyly in cattle as none of these sequence variants was observed in chromosomes from unaffected control animals.

The analysis of the syndactyly cases from the Holstein breed confirmed the presence of the recessively inherited c.4863_4864delCGinsAT mutation in eight cases, which were related to the recently identified founder cow named Raven Burke Elsie, born 1947 [9]. In contrast, four out of twelve syndactyly affected Holstein calves from our Holstein family I shared only a single, paternally inherited copy of this mutation. Therefore we assume the existence of further disease causing mutations within the *LRP4 *gene within the international Holstein population. Unfortunately, we did not find evidence for such a mutation in these four cases within in the 37 analyzed *LRP4 *exons. The identified exon 7, 29, and 32 SNP could be probably excluded as causative mutations for syndactyly since they did not alter the amino acid sequence and due to the observed high frequencies for the mutated alleles in the controls. As no genomic sequence data for the regulatory 5'region of *LRP4 *including exon 1 is publicly available, we were not able to analyze this gene region for possible mutations. There might be even another gene involved in the pathogenesis, e.g. modifying genes that would impact *LRP4 *expression. The bovine *ALX4 *gene, located within the mulefoot linked region on cattle chromosome 15, previously has been excluded in our affected animals as candidate gene [12].

The detected c.4940C>T SNP in Holstein family II co-segregates perfectly with the disease according to recessive inheritance and provides evidence that independent non-synonymous *LRP4 *mutations occur within the Holstein population, The two missense mutations in *LRP4 *which co-segregate with syndactyly in the Simmental family underline the identified allelic heterogeneity at the bovine *LRP4 *gene. The observed perfect co-segregation of each of these mutations within the analyzed families provides evidence that these mutations are potentially causative for syndactyly. All available parents carried a single mutated allele and the affected animals showed two copies of mutated alleles, respectively. Finally, the *LRP4 *genotypes of the affected animals from the crossbred family illustrate the existence of compound heterozygotes carrying two different deleterious *LRP4 *alleles. Due to records from the breeder, we assume that the dam of the affected sire II-1 (Figure 4A), a Holstein cow, had a well known syndactyly carrier sire (Marathon) in her ancestry. This could explain the occurrence of the c.4863_4864delCGinsAT mutation within this family.

**Figure 5:**
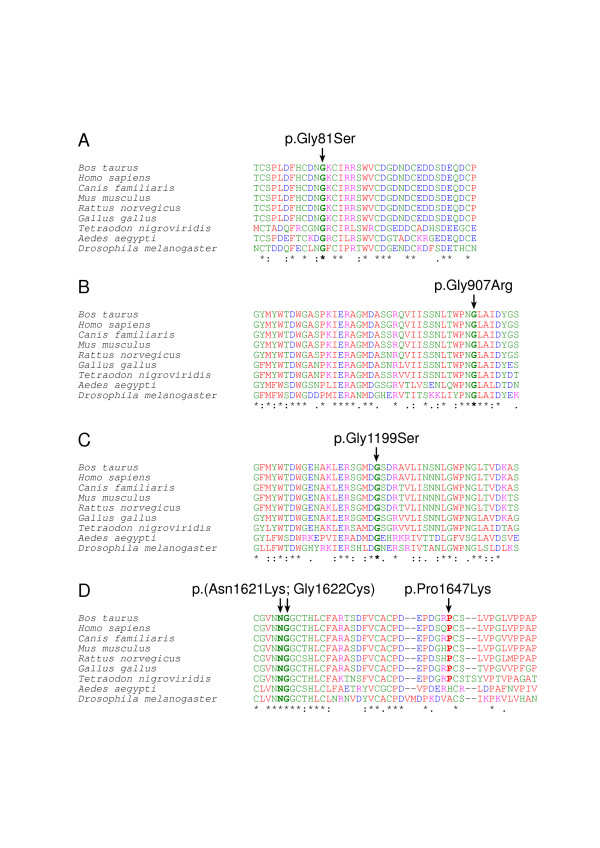
**Multispecies protein sequence alignment of four parts of LRP4**. The observed mutations are indicated by arrows and the respective residues are indicated in bold characters. Conserved cysteines (A+D) and YWTD motifs (B+C) are highlighted in grey. Protein sequences accession numbers used for the alignment: *Bos taurus *(ABE73152), *Homo sapiens *(NP_002325), *Canis familiaris *(XP_540748), *Mus musculus *(NP_766256), *Rattus norvegicus *(NP_112612), *Gallus gallus *(XP_421114), *Tetraodon nigroviridis *(CAF99960), *Aedes aegypti *(EAT37281), *Drosophila melanogaster *(NP_727914). Small and hydrophobic amino acids are indicated in red, acidic amino acids in blue, basic amino acids in magenta, and hydroxyl-, amine-, basic-Q amino acids in green, and others in gray, respectively. Identical residues are indicated by asterisks beneath the alignment, while colons and dots represent very similar and similar amino acids, respectively.

**Figure 6:**
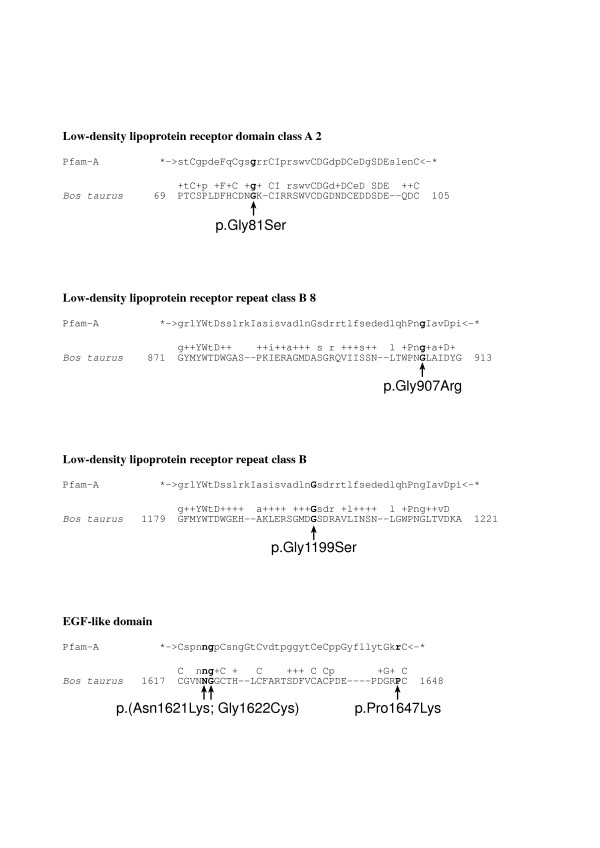
**Pfam alignments of conserved low density lipoprotein receptor domains class A, B and EGF like domains against the bovine LRP4 protein domains**. The observed mutations are indicated by arrows and the respective residues are indicated in bold characters. Pfam alignment consensus (Pfam-A) is given on the top and the respective bovine LRP4 segment indicated by numbers according to protein accession ABE73152 is given at the bottom. In between the alignment the most strong and strongly conserved amino acids are indicated as capital and small letters, respectively, and the + indicates the less conserved residues.

LRP4 represents a core member of the LDL receptor family which consists of seven structurally closely related transmembrane proteins [7]. All receptors are anchored in the plasma membrane by a single transmembrane domain and contain short cytoplasmic tails and the extracellular domains consist of a variable number of ligand binding-type repeats, always followed by EGF homology domains [7]. The LDL receptor represents the founding member of the family and over 700 independent mutations that disrupt the function of LDL receptor and cause familial hypercholesterolemia have been found [13]. Therefore we also compared the mutations we have found in the bovine LRP4 protein to those known in human LDLR. The four newly described *LRP4 *mutations in this article represent missense mutations, like the *LRP4 *exon 33 two nucleotide substitution reported before [9]. All four amino acid exchanges affect functionally important and particularly conserved extracellular domains of the bovine LRP4 protein (Table 1). An alignment of protein sequences of the four relevant homologous LRP4 domains from different species including mammals, birds, bony fishes and flies is shown in Figure 5. Furthermore, we used Pfam, a database of multiple sequence alignments and hidden Markov models covering many common protein domains and families [14,15], for alignments of the bovine LRP4 protein sequence against conserved low density lipoprotein receptor domain class A, B and EGF like domains with the Pfam search by protein sequence option using default parameters (Figure 6). To test possible functional consequences, the modified proteins were analyzed with two different software packages, PolyPhen [16,17] and SIFT [18,19] (Table 1).

The p.Gly81Ser mutation affects an invariably conserved glycine in a ligand binding LDL receptor class A domain (Figures 5 and 6), characterized by successive cysteine-rich repeats of about 40 amino acids, which occur in low density lipoproteins and related receptors [7]. Furthermore, it is highly likely that this mutation disrupts LRP4 function, as for example a frameshift mutation affecting residue 98 of human LDLR class A domain causes hypercholesterolemia in a Japanese family [20].

Two mutations (p.Gly907Arg, p.Gly1199Ser) affect low density lipoprotein receptor class B, or alternatively termed YWTD, domains which are found as multiple tandem repeats building the characteristic beta-propeller structure in low density lipoprotein receptors [7]. The p.Gly907Arg mutation affects a conserved glycine in LDL receptor class B domain 8 and the p.Gly1199Ser affects an invariably conserved glycine in LDL receptor class B domain 13 (Figures 5 and 6). A substitution of a valine for a glycine at residue 544 of human LDLR causes hypercholesterolemia and functional analysis of this mutation gives rise to an LDL receptor that is not transported to the cell surface and is rapidly degraded [21]. Several mutations of the human *LDLR *gene causing hypercholesterolemia affecting the LDL receptor class B domain have been reported [13]. Therefore it is likely that these two bovine mutations impair LRP4 function.

The p.Pro1647Lys mutation is located within a particular type of extracellular EGF-like motif, termed LDL-type EGF-like, which is characteristic for low density lipoprotein receptor related proteins [22]. This domain is also affected by the previously reported Holstein c.4863_4864delCGinsAT mutation [9] and the majority (47%) of *LDLR *mutations causing hypercholesterolemia in man were found in the EGF-like domain [23]. Additionally, the p.Pro1647Lys mutation may be dysfunctional due to the software predicted consequences (Table 1).

In summary, these comparisons provide support for the probable causality of the four newly identified bovine *LRP4 *mutations affecting codons 81, 907, 1199 and 1647, respectively. The three silent exonic mutations identified in the Holstein family I are very likely not causative, particularly as they occur at a rather high frequency in unaffected control animals. Finally, the published exon 33 substitution of two consecutive nucleotides could be confirmed in some of the affected Holstein calves and the recently reported *LRP4 *single nucleotide substitution at the first base of intron 37 (c.5385+1G>A) observed in two syndactyly affected Angus cattle was not observed in any of the examined cases within this study.

## Conclusion

We confirmed a significant role of *LRP4 *mutations in the pathogenesis of congenital syndactyly in cattle. This represents the third *LRP4 *report of mutations in cattle while the first two *LRP4 *mutations were described recently in Holstein and Angus cattle, respectively [9,10]. The data indicate that extensive allelic heterogeneity exists in cattle and within the Holstein breed. However, the four newly described *LRP4 *mutations do still not explain all analyzed cases of syndactyly. Therefore genetic testing for the carrier status of single individuals remains difficult, because at present obviously not all causal mutations have been detected. Thus, only presence of a known mutated allele can be recorded and unequivocal carriers are detected. Further studies of the regulatory 5'-region and exon1 of the bovine *LRP4 *gene will help to clarify if these regions are involved in the development of syndactyly.

## Methods

### Subjects

We analyzed a total of sixteen affected animals and samples of sixteen available relatives from three cattle populations (Holstein, Simmental and Holstein × Simmental × Charolais crossbred) with the clinical diagnosis of congenital syndactyly. The clinical diagnosis was confirmed by radiography. In a previous linkage study we reported eight affected German Holstein calves belonging to a single eight generation family, named Holstein family I [3]. This pedigree was extended by the inclusion of three cases of syndactyly in Italian Holstein and a single affected male German Holstein calf (VIII 1–4; Figure 1A). Each of these four cases could be maternally and paternally traced back to the single common male ancestor of the established Holstein family I. Additionally, a second German Holstein pedigree, named Holstein family II, with a single affected female calf without any relationship for five generations to family I was analyzed. Finally, a single affected purebred female German Simmental calf (Simmental family) and two affected crossbred bulls (father and son) belonging to a Holstein × Simmental × Charolais pedigree (crossbred family) were examined. All control samples used to check the distribution of sequence alterations were taken from an archive of unrelated artifical insemination sires matching to the German Holstein (n = 48), German Simmental (n = 48) and German Charolais populations (n = 16), respectively.

### Mutation analysis

Genomic DNA was isolated from blood samples by standard methods. The protein coding *LRP4 *exons 2 to 38 with the intronic splice site junctions were PCR amplified with primers described before [9]. The subsequent re-sequencing of the PCR products was performed after Shrimp Alkaline Phosphatase (Roche, Basel, Switzerland) and Exonuclease I (N.E.B., Axonlab, Baden, Switzerland) treatment using both PCR primers with the ABI BigDye Terminator Sequencing Kit 3.1 (Applied Biosystems, Rotkreuz, Switzerland) on an ABI 3730 capillary sequencer (Applied Biosystems). Sequence data were analyzed with Sequencher 4.6 (GeneCodes, Ann Arbor, MI, USA).

## Abbreviations

ALX4: aristaless-like homeobox 4

EGF: epidermal growth factor

GJA1: gap junction protein alpha 1

HOXD13: homeo box D13

LDL: low density lipoprotein

LDLR: low density lipoprotein receptor

LRP4: low density lipoprotein receptor-related protein 4

Megf7: multiple epidermal growth factor-like domains 7

OMIA: online mendelian inheritance in animals

OMIM: online mendelian inheritance in man

SIFT: sorting intolerant from tolerant

Pfam: protein family and domain database

PolyPhen: polymorphism phenotyping

## Authors' contributions

CD carried out the molecular genetic studies and drafted the manuscript. TL assisted in the molecular genetic analysis. IT, BH, OD, MH and AG made major contributions to the collection and phenotype examination of affected calves. AD and AE provided the PCR primers.
